# Trends in Childlessness Among Highly Educated Men in Sweden

**DOI:** 10.1007/s10680-018-9511-3

**Published:** 2019-01-17

**Authors:** Margarita Chudnovskaya

**Affiliations:** Stockholm University, Stockholm, Sweden

**Keywords:** Education, Fatherhood, Family formation, Childlessness

## Abstract

Among men with post-secondary degrees in Sweden, one in four are childless by age 45, and this level has been constant over time (in this study, for men born 1956–1972). This high level of childlessness is somewhat surprising in the context of a significant gender imbalance among the highly educated (and thus the relative scarcity of highly educated men). In this study, I examine differences in childlessness among the highly educated by studying how educational prestige, social class, and income are associated with the likelihood of becoming a father. Higher income and social class background are positively associated with fatherhood, and this association has not changed over time. Educational prestige (higher degrees, or degrees from traditional universities) is not positively associated with fatherhood, while 2-year degrees have become more positively associated with fatherhood over time. The findings of this study suggest that socioeconomic resources are important for men's family formation in Sweden compared to educational resources, contrary to expectations from educational homophily and partner market perspectives.

## Introduction

In most European countries, men with a post-secondary education are more likely to become fathers than men with a lower educational attainment (Burkimsher and Zeman 2017; Kravdal and Rindfuss 2008; Martín-García 2008; Nisén et al. 2017; Trimarchi 2016; Trimarchi and Van Bavel 2017). Despite the positive association between higher education and fatherhood, a substantial share of all highly educated men remain childless: about 20–25% in countries across Europe including Austria, Germany, the Netherlands, UK, Switzerland, Czech Republic, Bulgaria, Norway, Finland, and Sweden (Jalovaara et al. 2017; Miettinen et al. 2015). Importantly, the share of highly educated men remaining childless and/or single has remained steady or increased slightly across Europe over the last few decades (Becker and Jann 2017; De Hauw et al. 2017; Jalovaara et al. 2017; Wiik and Dommermuth 2014).

This persistence of rather high childlessness among highly educated men is at odds with predictions from theories of educational homophily and the partner market framework. Research on educational homogamy has shown that the highly educated tend to partner with each other. While opportunities play a role, the literature has stressed the importance of preferences for shared experiences, tastes, values, and intellectual orientation among the highly educated (Blossfeld 2009; Domański and Przybysz 2007; Kalmijn 1994; Mare 1991; Mäenpää 2015; Smits 2003). The ability of highly educated men to find highly educated female partners has increased dramatically over the last decades. Whereas systems of higher education were previously dominated by men, they are now dominated by women in Sweden and most other OECD countries (Vincent-Lancrin 2008; UKA 2016). Highly educated men are increasingly scarce on the partner market due to gender inequality in higher education. Previous research suggests that groups which are “in-demand” on the partner market benefit in terms of union formation (Abramitzky et al. 2011, Lewis and Oppenheimer 2000). Thus, relative scarcity should facilitate the formation of childbearing unions among highly educated men—yet highly educated men remain childless to a significant degree. Meanwhile, childlessness among highly educated women has decreased over this time period (Jalovaara et al. 2017 for the Nordic countries). Meanwhile, women in the Nordics and around the world (Esteve et al. 2012, 2016) increasingly “partner down” with men who have lower education than themselves.

The aim of this study is to promote our understanding of childlessness by assessing the extent to which childlessness among highly educated men is linked to socioeconomic status (SES) disadvantage. The contribution of this study is to answer the following two research questions: *How are measures of socioeconomic advantage related to childlessness among highly educated men? Has the association between socioeconomic advantage and childlessness weakened over time?* I use high-quality Swedish register data for men born 1945–1972, which provides complete and reliable records of biological fatherhood and socio-demographic variables, to address the relevance of social class background, educational prestige, and post-graduation income for childlessness.

## Understanding Childlessness Among Highly Educated Men

After critiques of traditional studies of childlessness which focused exclusively on women, much recent research attention has explored the transition to fatherhood (Balbo et al. 2013; Bledsoe et al. 2000; Forste 2002; Kreyenfeld and Konietzka 2017; Tanturri et al. 2015). This research shows that for men across Europe, inequalities in the transition to fatherhood are mediated by the inequalities in union formation; those men who remain childless often also remain un-partnered (Barthold et al. 2012; Jalovaara and Fasang 2017; Keizer et al. 2008; Miettinen 2010; Trimarchi and Van Bavel 2017; Schytt et al. 2014).

A percentage of all men will remain childless regardless of the partner market conditions, including men who prefer to live in same-sex partnerships, as well as men who face mental or physical health limitations which preempt family formation. However, this group is just a share of all childless men: A survey study from Finland suggests that less than 10% among all men childless by age 44 desired zero children (Miettinen 2010), and a similar figure is reported by Hakim using Family and Fertility Survey data covering 21 European countries (Hakim 2004). The majority of men are thus open to the option of fatherhood. Below I discuss how the partner search and resource perspective can be used to understand inequalities in the transition to fatherhood, and advance some competing explanations that would suggest socioeconomic status may not be as important as other factors.

### The Partner Market and the Resource Perspective

The partner search framework is the key demographic paradigm for understanding the formation of unions. According to this framework, individuals are (passively or actively) searching for a partner to enter a childbearing union, and they are presented with options from different environments (neighborhoods, schools, places of work and leisure) (Blau 1994; Kalmijn and Flap 2001). People may struggle to assess the quality of potential partners, and they also struggle to predict whether future options will be more attractive. Thus, individuals use some simple heuristics within the partner search and aim to “satisfy” some criteria in potential partners, such as age range, resources, personality, and appearance (Blossfeld and Timm 2003). The partner search process is double-sided: Individuals must choose partners and then be chosen in return. The partner search framework suggests that we can understand childlessness in two ways. Some men are unable to attract desirable partners, while some men are not interested in forming a union with partners available to them.

The resource perspective can help explain why some men are unable to attract partners. Men with higher socioeconomic status are more likely to be fathers (Barthold et al. 2012; Fieder and Huber 2007). This trend is classically explained by Becker’s (1993) *New Home Economics* model of household specialization, as women choose male partners who provide the highest standard of living for their families. Since the midcentury, European societies (and the Nordic countries in particular) have moved to a dual-earner family model where women are increasingly self-sufficient and their own resources increasingly matter in the partner search (Blossfeld and Drobnic 2001). Factors such as personality or gender-egalitarian attitudes increasingly matter for union formation (Goldscheider et al. 2015; Oláh and Bernhardt 2008; Skirbekk and Blekesaune 2014). Despite this shift, men’s resources continue to play an important role in union formation even within the egalitarian Nordic context (e.g., Lappegård et al. 2011; Hart 2015 for Norway; Nisén et al. 2017 for Finland; Silva 2016 for Sweden).

Higher education is one such desirable status position for men and is associated with lower levels of childlessness in many developed economies.^1^ However, the group of highly educated men is diverse in terms of additional socioeconomic status attributes. From a partner search framework, we expect that, just as education generally facilitates transition to fatherhood, the possession of other socioeconomic status attributes is also positively related to fatherhood. We can thus understand childlessness among highly educated men by considering inequalities within the group. The first hypothesis in this study is that *Socioeconomic status is negatively associated with childlessness among highly educated men.* Following the partner search theory and the resource perspective, men with higher socioeconomic status positions are more likely to be able to find a desirable partner and to form a childbearing union.

### Alternative Pathways to Childlessness

Contrary to the resource perspective, we might expect that among highly educated men, there are only insubstantial SES differences in fatherhood outcomes. One explanation could be that in the partner search process, higher education is a desirable status attribute—more important than other measures of SES. As shown by recent experimental research from online dating, highly educated women tend to select partners on educational similarity (Skopek et al. 2010; Hitsch et al. 2010a, b). According to theories of educational homogamy, homogamy is driven partially by a preference for economic resources, but also to the cultural value of education tied to knowledge, experiences, values, and networks cultivated in higher education (Blossfeld 2009; Kalmijn 1994). The resource perspective suggests that men with high SES would be advantaged in family formation—but if women also value the cultural, social, and knowledge capital of higher education, we would observe a weaker association between SES advantage and fatherhood among highly educated men.

**Fig. 1:**
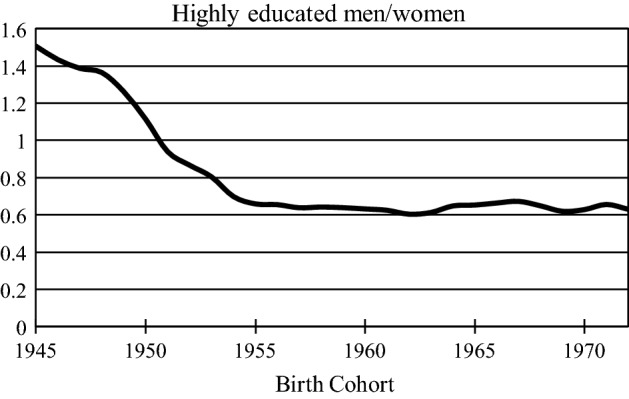
Sex ratio among highly educated men and women in Sweden by birth cohort, calculated from Swedish registers

A preference for a highly educated partner, regardless of their other SES attributes, would particularly be apparent because the sex ratio among the highly educated is heavily unbalanced. Figure 1 shows the sex ratio among men and women in Sweden who have a post-secondary degree at age 30, by birth cohort: Since the 1955 cohort there has been three highly educated men for five highly educated women.^2^ If many highly educated Swedish women strongly prefer highly educated partners, in the context of the unbalanced sex ratio, highly educated men would be desirable in the partner market, even if they have lower status according to other measures of SES. The Swedish welfare state and dual-earner family model may additionally facilitate such a pattern of partner choice. Most Swedish families are not solely reliant on the man’s income, and the transition to parenthood is financially facilitated through the provision of nearly free childcare, health care, and generous parental leave. Thus, if highly educated Swedish men are desirable due to their educational attainment, the unbalanced sex ratio would lead to lesser SES status differentiation among highly educated men in the transition to fatherhood.

An additional reason for a weak association between socioeconomic status and fatherhood would be if a share of highly educated men across the SES spectrum have preferences for a *childfree* lifestyle focused more on development of self, career, or non-reproductive family (Lesthaeghe 1995; Tanturri and Mencarini 2008; Van de Kaa 2001). Such post-materialist values and non-traditional lifestyles have been associated with highly educated groups, and it could be the case that highly educated men—regardless of, e.g., income, or social class background—prefer to live without a partner or children. This may particularly be apparent in Sweden, as the social acceptance of childlessness is relatively high in the Nordic countries (Merz and Liefbroer 2012). However, studies of educational differences in fertility desires suggest education is positively linked with fertility desires (Berrington and Pattaro 2014). Another potential explanation could be that the over-supply of highly educated women implies a lower willingness among men to commit to any partner and thus to become fathers. According to a hypothesis from Guttentag and Secord (1983), highlighted by De Hauw et al. (2017), when the pool of potential partners and unexplored relationship opportunities is large, it may be difficult for individuals to make a choice about a suitable partner and thus to end the partner search. Following such a theory, the most advantaged men would have an over-abundance of choice and thus avoid fatherhood.

### Multiple Measures of Socio-Economic Status

As presented above, there are compelling reasons to believe that SES may or may not be strongly related to fatherhood among highly educated men. This study measures SES in three different ways, the first of which is *educational prestige.* Education matters not only because it captures information about future income or career prospects, but because of the specific cultural value that higher education has for individuals (Bruze 2011; Skopek et al. 2010). By completing a higher education degree, individuals participate in a process of intellectual and personal development; they acquire cultural knowledge, as well as social knowledge and access to social networks of other highly educated individuals. The cultural and intellectual resources might be particularly important for other highly educated individuals, who seek a match in terms of cultural capital (Kalmijn 1994), but such resources should generally be an asset in the partner search process. To the extent that educational resources are an attractive asset for men, we would expect that educational prestige (here captured by length of degree and institutional classification) would correspond negatively to childlessness.

The second SES measure considered is *social class background*. Sweden is a country with a relatively equal education system (Breen and Jonsson 2005), and yet higher education and social class are positively associated. However, the social benefits of higher education accrue differently to individuals of different class backgrounds. Students from lower class backgrounds may be less able to take advantage of the social environment and form networks and relationships as demonstrated by studies within the US context (Armstrong and Hamilton 2013; Brand and Davis 2011; Musick et al. 2012). Additionally, men from higher class backgrounds have access to economic, social, and cultural resources from their family, which may be an additional asset in partnership formation. We would thus expect that highly educated men who come from a lower class background would have a higher probability of remaining childless.

The final SES measure included in this study is *income*. Income is an important determinant of family formation because of the expenses associated with parenthood. Income is also correlated with desirable individual and personality factors. Men who enter relationships in Sweden tend to increase their income (Regnér and Isacsson 2008), and men who want to become fathers are likely find jobs which offer them the necessary financial opportunities. Additionally, men who were able to complete a higher education but who end up in a low income position may be less motivated to become fathers, or may be less attractive as potential partners. We would thus hypothesize that for highly educated men, higher income is negatively associated with remaining childless.

## Trends Over Time

The second research question in this study is: *Has the association between socioeconomic status and childlessness weakened over time?* This study population includes men born 1945–1972, who became fathers in the years 1970–2010, and thus captures the time period with major social changes in Sweden toward gender egalitarianism (Oláh and Bernhardt 2008). Following the initial hypothesis, we would expect that men who had a lower socioeconomic status position would be more likely to remain childless, but there are two reasons why such status attributes may have weakened in their association with fatherhood over the time period studied.

Firstly, there have been some changes in the availability of male partners over time. Figure 1 presented earlier shows the sex ratio among the highly educated over time: Highly educated men were in abundance in cohorts born 1945–1950, but scarce in cohorts born 1950–1972. Following partner market logic, we would expect that other socioeconomic resources mattered more when highly educated men were abundant. Increasing competition among women could mean that women are willing to compromise some of their other preferences (like income) to find a highly educated partner.

Secondly, Swedish women during this period have increased their labor force participation and income, and unions have become increasingly gender-egalitarian. Thus, it is possible that when choosing among highly educated male partners, women’s preferences for partners with a high socioeconomic status have weakened as women instead focus on personality factors, values, or attitudes toward relationships and housework (Press 2004). During the time period covered by this study, European societies have undergone a values shift, as the emphasis on tradition has decreased and an emphasis on individual choices have increased (Giddens 1992). From this perspective, resources may play a weaker role in predicting the transition to fatherhood because unions are formed on non-material concerns, or without regard for men’s traditional roles as financial providers. The extent to which individuals increasingly value emotional connection or non-material concerns in relationships within the Nordics remains understudied. Research among recent young cohabiters in Sweden and Norway provides some mixed support for such a theory: The results suggest that income is less relevant as a factor for plans to marry than emotional considerations, though education remains strongly associated with marriage plans (Wiik et al. 2010). Nevertheless, it is possible that in the transition to fatherhood, factors such as social class background or income have become less important over time due to changes in the structure of the partner market and due to changes in values. The second hypothesis in this study is thus that: *The relevance of socioeconomic status attributes for childlessness among highly educated men has diminished over time.*

It is possible that the association between childlessness and SES would not diminish over time. An explanation would be that, in the partner search process, higher education matters less than other indicators of socioeconomic status. Thus, some men are consistently unable to find a partner due to socioeconomic status constraints, regardless of their educational level. Attributes such as income may be more important in the partner selection process, or more strongly associated with the desire for family formation than higher education. Such a conclusion may seem to be at odds with the emphasis on the desirability of highly educated men. However, it is important to note that through the process of educational expansion, increased horizontal inequalities have emerged within the highly educated group. Highly educated men have generally become “more average” (Wiik and Dommermuth 2014), as have highly educated women. The cultural value of higher education in the partner search in Sweden may not be as significant as other status attributes when the group of highly educated people is more heterogenous, and the extent to which higher education is a marker of shared values and experiences may have also changed.

## Data and Method

The study population includes all men born in the years 1945–1972. Men who have been registered as residents in Sweden from the year 1960 and onwards have a digitized personal identification number, which makes it possible to identify all highly educated men and link them to their family and socioeconomic status indicators. Men who immigrated to Sweden after age 15 are omitted from the study for the sake of complete childbearing and education records (this removed around 39,000 observations). Men who did not survive to the age of 40 are also omitted (this removed 1379 observations). Using the personal identification number, men are followed until 2012, the latest year for which data are available. Men are connected to their first child via the multi-generational register, which provides nearly complete information on men’s childbearing (Thomson and Eriksson 2013). Men’s education is taken from the higher education graduation register, and all men who have completed any post-secondary degree between the years 1962 and 2012 are in the study sample. This cohort selection is driven to some extent by data constraints (data are currently not available after the year 2012), but this choice also covers cohorts who began attending college during the initial stages of the educational expansion (launched in 1977), and until the 1990s. The outcome analyzed in this study is childlessness by age 40. In the cohorts studied, 6% of men become fathers after age 40 and 1% become fathers after age 45. Age 40 is used in the study to allow for the inclusion of the youngest cohorts, but sensitivity analyses show that this cutoff does not meaningfully change the results except with regard to differences in predicted childlessness by cohort.

A major strength of this study comes from the coverage afforded by register data. The nearly complete coverage in the multi-generational register ensures accurate coverage of fatherhood. The long time span allows for childlessness trends to be studied across decades, and factors such as social class background or income can be analyzed with great precision. However, register data also have a number of drawbacks which hamper the study of transition to fatherhood. Swedish registers do not have a dwelling register, and thus, it is not possible to identify cohabiting partnerships without shared biological children. This makes it impossible to control for partnership status or for the characteristics of the men’s partners (unless they have a child together). The absence of dwelling information also means that this study does not include any information on social fatherhood. Men may be living with a female partner who has children from a previous union, but as long as men are not biological fathers to a child, they will be classified as childless in this study. Finally, the registers can only provide one perspective on this puzzle, as we lack information on men’s attitudes and preferences which might be available in a survey. Despite these constraints, this study offers a significant step in promoting our understanding of childlessness among highly educated men.

Three SES indicators are included in this study. The first is educational prestige. This variable is a combination of the length of the study degree and the quality of the educational institution. First, I differentiate men based on whether they have a short post-secondary degree (up to 2 years), compared to whether they have a bachelor’s (3 years) or a master’s (4/5 years) or professional (medicine, architecture) degree. Additionally, I differentiate between men who studied at traditional institutions of study, compared to men who studied at institutions which were opened or upgraded following educational expansion policy enacted in Sweden in 1977 (Premfors 1984).^3^ Newer institutions were opened throughout the country to be accessible to students outside major cities, while traditional institutions are located in historic student towns. They not only have an academic prestige factor but have offered a more traditional student environment and intensive social experience, which may facilitate partnership formation and the eventual transition to fatherhood. This variable thus consists of the following options: 2-year degree, any degree at newer institution, bachelor’s degree at traditional institution, and master’s/professional degree at traditional institution.

The second factor included is social class background. This measure is drawn from census data on the men’s parents when the men in the study are aged 10–15. I use census data from 1960, 1970, and 1980 and convert Statistics Sweden’s SEI measure to an EGP class measure with seven levels: Upper service class, lower service class, routine non-manual workers, small employers and entrepreneurs, lower-grade technicians, skilled working class, and unskilled working class. This class measure reflects the Erikson–Goldthorpe class divisions, and I use the highest position observed among the man’s mother and father (Erikson 1984; Erikson and Goldthorpe 1992). A small percentage of all men are missing information on class background (about 5%), and these are also included in the model. In these cases, the man’s parents are present in Sweden, but neither parent has a classifiable occupation within any appropriate census year.

The third factor examined in this study is income, from the Income and Taxation register spanning 1968–2012. The measure is “disposable income,” which includes work income and many different kinds of benefits, and thus captures the total financial resources at an individual’s disposal. I use men’s disposable income 5 years after graduation from their first post-secondary degree and compare it to the distribution of all incomes earned by all men who are in their age group (± 2 years), in that year, regardless of educational category. Income is ranked in quintiles from 1 (lowest) to 5 (highest). Although men who plan to form a family and those who become fathers also achieve a higher income, comparing men 5 years after graduation limits the scope of this effect to some extent. This measure also provides a good comparison of men’s resources at a time when they would be seeking a partner or preparing for fatherhood.

The year of birth is included in the models to examine the time effect, and for ease of presentation and interpretation I group birth cohorts into 5-year groups: 1945–1949, 1950–1954, 1955–1959, 1960–1964, 1965–1969, and 1970–1972. I also include a dummy variable for those born outside Sweden, but arriving prior to age 15 (about 2% of the study population).

The statistical method employed in this study is binomial logistic regression with the outcome “Childless by age 40.” The logistic regression results are presented as odds ratios, where an odds ratio of greater than 1 suggests a greater likelihood of childlessness than the reference category and odds ratios of less than 1 represent lesser likelihood. Logistic regression is used rather than an event-history framework because logistic regression results are more intuitive in interpreting the likelihood of ever transitioning to fatherhood than an event-history intensity of transition approach. Similar results were found when estimating a linear probability model and when experimenting with different age cutoffs (and these results are available upon request). To answer the research question about change over time, I test the interaction between the birth cohort variable and socioeconomic status covariates. The results of the interaction models are estimated using Stata (Williams 2012) and presented as marginal effects figures which show the likelihood of being childless at age 40 in relation to the birth cohort group and the SES covariate being examined, in addition to the full model specification with all covariates set to their reference categories.

## Descriptive Statistics of Study Population

Table 1 shows the composition of the study population, broken down by cohort group. Stability is the key take away from this table. The total number of men in each cohort group is roughly similar, except the latest cohort group, which includes only 3 years. By age 40, about 29% of men are childless, and the percentage childless by age 45 is relatively constant at 25% (with a slight decrease among the youngest cohort). The mean age at graduation (26–27) and mean age at first birth (31–33) are also relatively constant, with a slight increase over time.

**Table 1:** Descriptive characteristics of the study population

	1945–1949	1950–1954	1955–1959	1960–1964	1965–1969	1970–1972
Study *N*	31,006	29,887	32,296	31,595	37,352	26,382
% Childless by age 40	28	29	28	30	29	26
% Childless by age 45	25	25	24	25	24	–
Mean age graduation	26	27	27	27	27	27
Mean age first birth	31	32	32	33	33	33
Swedish born	98	98	98	98	98	97
*Class background (%)*						
Upper service	18	16	20	23	30	28
Lower service	25	27	32	32	27	29
Routine non-manual workers	12	12	11	10	7	7
Entrepreneur	10	8	7	6	8	7
Lower-grade technician	7	7	5	4	3	3
Skilled working	12	14	12	13	9	10
Unskilled working	11	12	10	10	10	11
Not available	5	4	3	3	6	5
*Educational prestige (%)*						
2 years	17	25	30	28	24	19
3 + years, newer	2	7	10	12	17	26
3 years, traditional	55	37	25	22	20	17
4 + years, traditional	27	31	35	39	39	38
*Income quintile (%)*						
1 (lowest)	5	5	5	5	5	5
2	8	11	11	11	11	9
3	13	20	21	20	19	20
4	26	29	29	29	28	30
5 (highest)	49	36	34	36	38	37

The sample is dominated by individuals with an advantaged class background: About 40% in older cohorts come from service class families and more than 50% among younger cohorts. In terms of educational prestige, the mix changes slightly across all cohorts. Newer institutions were not accessible to older cohorts but increasingly became popular, while the share of men graduating from traditional institutions with short degrees declined and the share graduating with longer degrees increased. In terms of income, these is also a stable trend. Graduates from the 1945–1949 cohort were especially likely to have earnings in the highest income quintile, but overall few highly educated men have low earnings compared to all men in their age group. About half of the highly educated men earn within 40–80% of the income distribution of men their age, and only 5% of highly educated men consistently fall into the lowest income quintile.

## Results

The results of the logistic regression model for all cohorts are presented in Table 2. The model includes a control for birth cohort and being born outside Sweden, and variables for social class origin, educational prestige, and income 5 years after graduation. All variables are included simultaneously in the model, and results are very similar to a stepwise specification (available on request). Results are reported in odds ratios. Because the study captures the entire population of highly educated men in Sweden, *p* values are not reported, though 95% confidence intervals are provided. There is not a clear cohort trend over the majority of the period, as reflected in the descriptive table, with the exception of the lower childlessness among the 1970–1972 cohorts.

**Table 2:** Results of logistic regression: outcome variable childless at age 40

Variable	Estimate (OR)	95% Confidence interval
*Birth cohort group*		
1945–1949	1.00	
1950–1954	0.98	[0.95, 1.03]
1955–1959	0.94	[0.91, 0.98]
1960–1964	1.03	[1.00, 1.07]
1965–1969	1.00	[0.97, 1.04]
1970–1972	0.82	[0.78, 0.87]
Not Swedish born	1.13	[1.05, 1.21]
*Social class background*		
1. Upper service	1.00	
2. Lower service	1.06	[1.02, 1.08]
3. Routine non-manual workers	1.09	[1.04, 1.13]
4. Entrepreneur	1.08	[1.06, 1.16]
5. Lower-grade technician	1.14	[1.08, 1.20]
6. Skilled working	1.31	[1.26, 1.36]
7. Unskilled working	1.34	[1.29, 1.40]
8. Missing	1.27	[1.22,1.33]
*Educational prestige*		
2 year, any inst.	0.84	[0.81, 0.86]
3 + years, new inst.	1.24	[1.19, 1.28]
3 years, traditional inst.	0.99	[0.97, 1.03]
4 + years, traditional inst.	1.00	
*Income quintile*		
1 (lowest)	3.07	[2.93, 3.22]
2	2.19	[2.12, 2.28]
3	1.88	[1.82, 1.94]
4	1.51	[1.47, 1.55]
5 (highest)	1.00	
*Constant*	0.25	[0.24, 0.26]
N	188,518	

The hypothesis advanced above was that SES advantage was likely to be correlated with entry to fatherhood, and this hypothesis is partially supported by the results. For social class background, this hypothesis is supported: there is a positive association between social class and childlessness. Men from working-class backgrounds (and those whose parents are missing occupational information) are the most likely to remain childless compared to men from service class backgrounds. For income, men who are in the top quintile are those who are the most likely to become fathers. Furthermore, there is a substantial difference between every quintile of the income distribution. Overall, the combination of social class and income is very strongly associated with childlessness. Within the study population, 20% of men come from a service class background and rank within the top income quintile—one in five of these men with high socioeconomic status is childless. Among the 15% of men who come from a working-class background, and whose earnings fall into the “middle” quintiles (40–80% of the income distribution), one in three men is childless.

For educational prestige, the hypothesis is only partially supported. Men who attend newer institutions (one in four men among the very youngest cohorts) are more likely to remain childless than men who attend traditional institutions. However, there is not a substantial difference between men who earn shorter or longer degrees from traditional institutions. Additionally, men with the lowest educational prestige, those who have a short post-secondary education, have a substantially lower likelihood of remaining childless.

The logistic regression model pooled together men from the different birth cohorts. To examine whether the relationship between socioeconomic status and fatherhood has weakened over time, I performed interaction analysis between the cohort grouping and the status variables. The results include the full model from Table 2, as well as cohort group–covariate interaction terms, with all other covariates set to the reference category. These results are shown as a series of marginal effects figures.

**Fig. 2:**
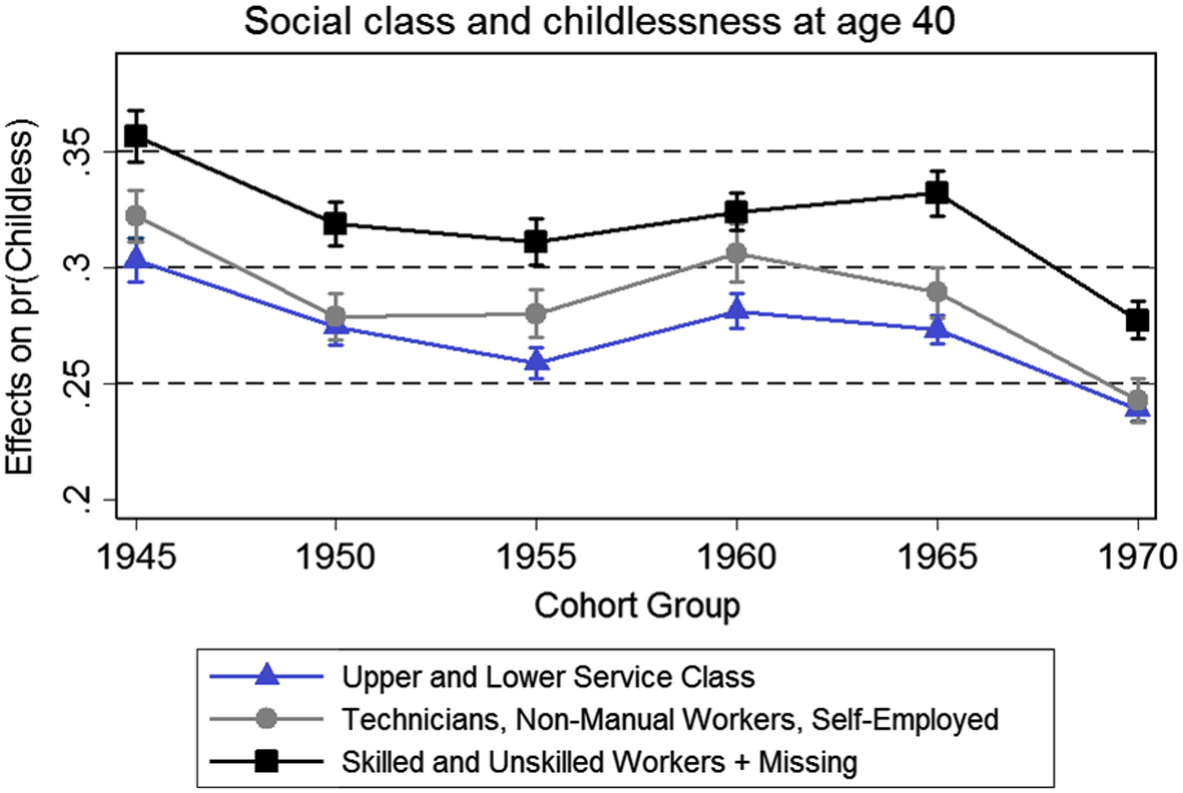
Results of interaction analysis: social class and childlessness (model also includes all covariates from Table 2, estimate with all covariates set to reference categories)

The first result (Fig. 2) shows the marginal effects of social class on childlessness for different cohort groups. To avoid a busy picture with eight different lines, the classes have here been collapsed into three groups: those whose parents were (1) in the upper and lower service class, (2) technicians, non-manual workers, self-employed, and small employers, and (3) skilled workers, unskilled workers, and those missing information. Generally, there is little evidence that social class has become a weaker predictor of childlessness over time, as shown by the non-overlapping confidence intervals. The main distinction is the difference between children from working-class families and children from all other families, and this difference in childlessness persists across all cohorts.

**Fig. 3:**
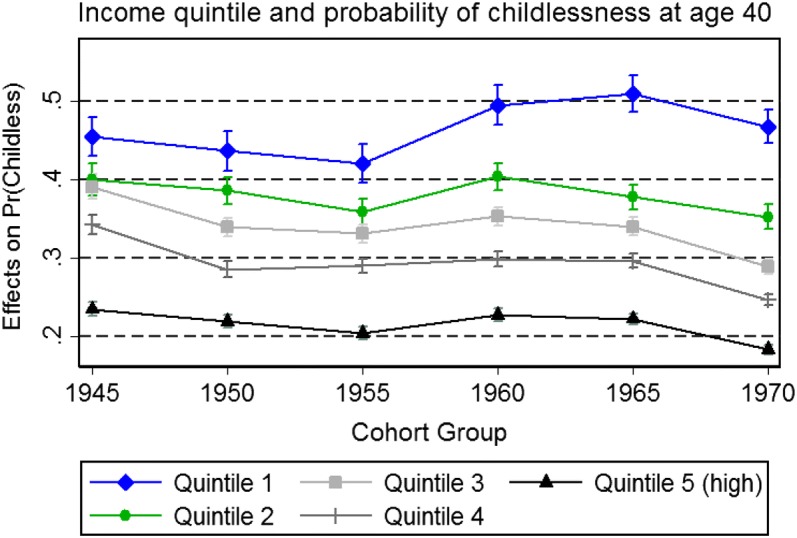
Results of interaction analysis: income and childlessness (model also includes all covariates from Table 2, estimate with all covariates set to reference categories)

The second interaction result (Fig. 3) is on the association between income and childlessness. This association also remains constant over time. The negative gradient in income and childlessness is clear across all cohorts, even though the predicted level of childlessness varies somewhat over time.

**Fig. 4:**
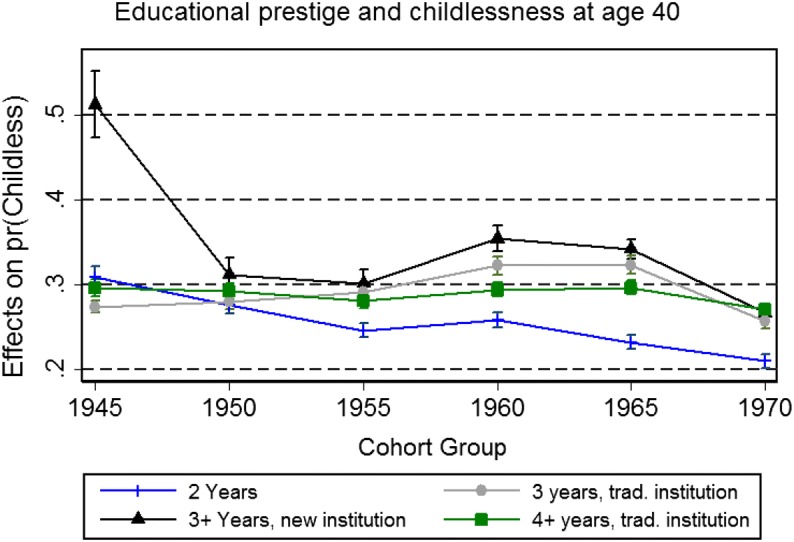
Results of interaction analysis: educational prestige and childlessness (model also includes all covariates from Table 2, estimate with all covariates set to reference categories)

The final result (Fig. 4) shows trends for the association between educational prestige and childlessness over time. Contrary to the findings on social class and income, the findings on educational prestige in relation to fatherhood over time are somewhat mixed. In terms of differences among university types, there are no important time trends. Graduation from newer institutions was highly associated with childlessness among the oldest cohort group, but this association is not significant for later cohort groups. This effect was likely observed because among the oldest cohorts, the group of men who were able to graduate from the newer post-secondary institutions was older at the time of completing their studies, and likely more negatively selected on grades and social background.^4^ One significant change is that the association between completing a short post-secondary education and fatherhood has strengthened over time. Men who graduate with these degrees are decreasingly likely to remain childless compared to men pursuing longer degrees.

## Discussion

Over the last decades, there has been a shift from an overrepresentation to an underrepresentation of men in post-secondary education, in Sweden and most OECD countries (Vincent-Lancrin 2008; UKA 2016). Due to this reversal in the gender imbalance, highly educated men have become relatively scarce in the partner market and thus following demographic theories of the partner market and educational homophily, we would expect a decline in childlessness within this group. However, recent research shows that childlessness among highly educated men in Europe has not declined over the last decades (De Hauw et al. 2017; Wiik and Dommermuth 2014).

This study investigates childlessness among highly educated men in Sweden, a country where the gender reversal in higher education occurred several decades ago. The strength of this study is high-quality Swedish register data, which makes it possible to examine all highly educated men born in the years 1945–1972 (including more than 188,000 observations). As in other European countries, childlessness among highly educated men has not declined in the birth cohorts studied—the prevalence of childlessness has been constant, with the exception of the very youngest cohorts examined (born 1970–1972).

Childlessness is a complex phenomenon with multiple explanations. This study suggests that in Sweden, compositional changes are unlikely to matter for understanding childlessness—roughly the same percentage of men have become highly educated across the cohorts studied, and the socioeconomic composition of the group is similar over time. Previous studies suggest that for European men, childlessness is typically associated with remaining un-partnered (Barthold et al. 2012; Jalovaara and Fasang 2017; Keizer et al. 2008; Trimarchi and Van Bavel 2017), and thus, a partner market perspective is helpful for understanding childlessness.

The partner market perspective implies that men and women who remain un-partnered are either unable to find a partner who meets their desired criteria, or unable to attract desired partners. Socioeconomic resources are known to be an important factor in the partner search process. Highly educated men possess one already valuable status attribute—their education—but other socioeconomic status attributes could explain differences in fatherhood outcomes. Thus, this study answers the following two research questions: (1) *How are measures of socioeconomic status (SES) advantage related to childlessness among highly educated men?* and (2) *Has the association between socioeconomic status advantage and childlessness weakened over time?*

Using Swedish register data, SES is measured in three different ways: *Social Class Background* (using a seven-class EGP classification), *Income* (measured as income relative to other Swedish men 5 years post-graduation), and *Educational Prestige* (as measured by post-secondary degree length and the type of institution attended). These socioeconomic status indicators are correlated, but they are independently associated with the childlessness outcome. The major limitation of this study is that union status is excluded from the study, and thus, it is not possible to study the transition to fatherhood process within cohabiting unions or to include partner characteristics in the models.

With regard to the first research question, the results of this study show that in general, SES advantage is associated with lower childlessness. In particular, higher social class background and income are associated with lower childlessness among highly educated men. Among the 20% of highly educated men who come from a service class background and earn within the highest quintile post-graduation, one in five remained childless. Among the 15% of highly educated men who come from a working-class background, and whose earnings fall into the “middle” quintiles (40–80% of the income distribution), one in three men is childless.

The strong income and class gradient observed is not completely intuitive in the Swedish context, which is characterized by a dual-earner family system, and a welfare state which facilitates family formation through generous subsidies and family policies. Despite the importance of the disadvantage gradient in childlessness, childlessness is relatively high even among men with a high SES. This result suggests that further research must consider additional explanations, including non-material factors important for partnership formation (e.g., values or attitudes toward home equality), preferences for voluntary childlessness, and the prevalence of social fatherhood (which cannot be captured in the registers).

An additional key result regarding the first research question was the absence of a childlessness gradient in educational prestige. For Swedish men, a professional degree (e.g., medicine, dentistry, or architecture) from a traditional university does not seem to be an asset in the formation of a childbearing union, compared to a bachelor’s degree from a regional college or a short post-secondary education. The absence of a clear gradient in childlessness with regard to educational prestige is interesting compared to income and social class. One explanation could be with regard to timing of fatherhood—the advantage of men with shorter post-secondary education is that they spend less time in education, and thus become fathers on average 2 years earlier (mean age 31 rather than 33). A plausible interpretation is that in Sweden, the weak distinction based on educational experiences reflects the general weakness of cultural/professional status hierarchies in society.

With regard to the second question, the results show that there have not been significant changes in the association between SES and childlessness in this group over time. The hypothesis in this study was that this association would weaken, based on the scarcity of highly educated men in the partner market, increased gender egalitarianism, and the spread of post-materialist values. There were some minor changes in educational prestige and childlessness over time, but the associations for income and social class remained consistent. Why was this trend consistent over time despite theoretical predictions based on partner availability and educational homophily? Future research is needed to understand this result. Perhaps the social value of education has weakened with educational expansion, and higher education is not an asset in the partner search compared to other forms of status. An additional explanation could be that the composition of highly educated women changed so that they no longer have a preference for highly educated men, and thus there is not a “high demand” for this group. An additional explanation could be that among highly educated men, lower income is correlated to personality or lifestyle factors related to a weak family orientation or low childbearing desires.

Additionally, future research would also be useful from a cross-national perspective, where there is greater inequality in prestige within the educational system (compared to Sweden, which is relatively non-hierarchical), or where there is a stronger social divide between the different educational groups. A register-based approach is also not able to measure attitudes and values and thus can only infer about the importance of resources versus preferences, so survey-based or qualitative research within the Nordic context would also be highly useful.

## Footnotes

1 Though the educational advantage in fatherhood is a common pattern in Europe, it is not a universal phenomenon as highly educated men have somewhat higher levels of childlessness than other men in some countries including France (Toulemon et al. 2008) and the UK (Berrington 2017).

2 The graph is a calculation from Swedish registers and shows the number of highly educated men born year x divided by the average number of highly educated women born in years* x* to *x* + 2, to account for standard age differences.

3 The traditional group includes the following institutions: Chalmers Technical College, Gothenburg University, Stockolm School of Economics, Karolinska Medical School, the Royal Institute of Technology, The Teacher’s College in Stockholm, Linköping University, Lund University, Stockholm University, the Swedish Agricultural University, Umeå University, and Uppsala University.

4 Most of these institutions started granting degrees in the late 1970s, meaning men would be close to 30 years in age when they enrolled.

## References

[CR1] Abramitzky R, Delavande A, Vasconcelos L (2011). Marrying up: the role of sex ratio in assortative matching. American Economic Journal: Applied Economics.

[CR2] Armstrong EA, Hamilton LT (2013). Paying for the party.

[CR3] Balbo N, Billari FC, Mills M (2013). Fertility in advanced societies: A review of research. European Journal of Population.

[CR4] Barthold JA, Myrskylä M, Jones OR (2012). Childlessness drives the sex difference in the association between income and reproductive success of modern Europeans. Evolution and Human Behavior.

[CR5] Becker G (1993). A treatise on the family.

[CR6] Becker R, Jann B (2017). Educational expansion and homogamy. An analysis of the consequences of educational upgrading for assortative mating in Switzerland. Swiss Journal of Sociology.

[CR69] Berrington Ann (2017). Childlessness in the UK. Demographic Research Monographs.

[CR7] Berrington A, Pattaro S (2014). Educational differences in fertility desires, intentions and behaviour: A life course perspective. Advances in life course research.

[CR8] Blau PM (1994). Structural contexts of opportunities.

[CR9] Bledsoe C, Lerner S, Guyer J (2000). Fertility and the male life cycle in the era of fertility decline.

[CR10] Blossfeld HP (2009). Educational assortative marriage in comparative perspective. Annual Review of Sociology.

[CR11] Blossfeld HP, Drobnic S (2001). Careers of couples in contemporary society: From male breadwinner to dual-earner families: From male breadwinner to dual-earner families.

[CR12] Blossfeld HP, Timm A (2003). Who marries whom? Educational systems as marriage markets in modern societies.

[CR13] Brand JE, Davis D (2011). The impact of college education on fertility: Evidence for heterogeneous effects. Demography.

[CR14] Breen R, Jonsson JO (2005). Inequality of opportunity in comparative perspective: Recent research on educational attainment and social mobility. Annual Review of Sociology.

[CR15] Bruze G (2011). Marriage choices of movie stars: does spouse’s education matter?. Journal of Human Capital.

[CR16] Burkimsher, M., & Zeman, K. (2017). Childlessness in Switzerland and Austria. In *Childlessness in Europe: Contexts, causes, and consequences* (pp. 115–137). Springer.

[CR17] De Hauw Y, Grow A, Van Bavel J (2017). The reversed gender gap in education and assortative mating in Europe. European Journal of Population.

[CR18] Domański H, Przybysz D (2007). Educational homogamy in 22 European countries. European Societies.

[CR19] Erikson R (1984). Social class of men, women and families. Sociology.

[CR20] Erikson R, Goldthorpe JH (1992). The constant flux: A study of class mobility in industrial societies.

[CR21] Esteve A, García-Román J, Permanyer I (2012). The gender-gap reversal in education and its effect on union formation: The end of hypergamy?. Population and Development Review.

[CR22] Esteve A, Schwartz CR, Bavel J, Permanyer I, Klesment M, García-Román J (2016). The end of hypergamy: Global trends and implications. Population and development review.

[CR23] Fieder M, Huber S (2007). The effects of sex and childlessness on the association between status and reproductive output in modern society. Evolution and Human Behavior.

[CR24] Forste R (2002). Where are all the men? A conceptual analysis of the role of men in family formation. Journal of Family Issues.

[CR25] Giddens A (1992). The transformation of intimacy: Sexuality, love and intimacy in modern societies.

[CR26] Goldscheider F, Bernhardt E, Lappegård T (2015). The gender revolution: A framework for understanding changing family and demographic behavior. Population and Development Review.

[CR27] Guttentag, M., & Secord, P. F. (1983). Too many women? The sex ratio question.

[CR28] Hakim C (2004). Childlessness in Europe.

[CR29] Hart RK (2015). Earnings and first birth probability among Norwegian men and women 1995–2010. Demographic Research.

[CR30] Hitsch GJ, Hortaçsu A, Ariely D (2010). Matching and sorting in online dating. American Economic Review.

[CR31] Hitsch GJ, Hortaçsu A, Ariely D (2010). What makes you click?—Mate preferences in online dating. Quantitative marketing and Economics.

[CR32] Jalovaara M, Fasang AE (2017). From never partnered to serial cohabitors: Union trajectories to childlessness. Demographic Research.

[CR33] Jalovaara, M., Neyer, G., Andersson, G., Dahlberg, J., Dommermuth, L., Fallesen, P., & Lappegård, T. (2017). *Education, gender, and cohort fertility in the Nordic countries*. Stockholm: Stockholm University, Department of Sociology (Stockholm research reports in demography 2017: 6).

[CR34] Kalmijn M (1994). Assortative mating by cultural and economic occupational status. American Journal of Sociology.

[CR35] Kalmijn M, Flap H (2001). Assortative meeting and mating: Unintended consequences of organized settings for partner choices. Social Forces.

[CR36] Keizer R, Dykstra PA, Jansen MD (2008). Pathways into childlessness: Evidence of gendered life course dynamics. Journal of Biosocial Science.

[CR37] Kravdal Ø, Rindfuss RR (2008). Changing relationships between education and fertility: A study of women and men born 1940 to 1964. American Sociological Review.

[CR38] Kreyenfeld M, Konietzka D (2017). Childlessness in Europe: Contexts, causes, and consequences.

[CR39] Lappegård T, Rønsen M, Skrede K (2011). Fatherhood and fertility. Fathering.

[CR40] Lesthaeghe R, Mason KO, Jensen A-M (1995). The second demographic transition in Western countries: An interpretation. Gender and family change in industrialized countries.

[CR41] Lewis SK, Oppenheimer VK (2000). Educational assortative mating across marriage markets: Nonhispanic whites in the United States. Demography.

[CR42] Mäenpää, E. (2015). Socio-economic homogamy and its effects on the stability of cohabiting unions. *Finnish Yearbook of Population Research*, *50*.

[CR43] Mare RD (1991). Five decades of educational assortative mating. American Sociological Review.

[CR44] Martín-García T (2008). ‘Bring Men Back In’1: A re-examination of the impact of type of education and educational enrolment on first births in Spain. European Sociological Review.

[CR45] Merz EM, Liefbroer AC (2012). The attitude toward voluntary childlessness in Europe: Cultural and institutional explanations. Journal of Marriage and Family.

[CR46] Miettinen A (2010). Voluntary or involuntary childlessness? Socio-demographic factors and childlessness intentions among childless finnish men and women aged 25–44. Finnish Yearbook of Population Research.

[CR47] Miettinen, A., Rotkirch, A., Szalma, I., Donno, A., & Tanturri, M. L. (2015). *Increasing childlessness in Europe: Time trends and country differences*. Stockholm University, Stockholm (families and societies working paper 33).

[CR48] Musick K, Brand JE, Davis D (2012). Variation in the relationship between education and marriage: Marriage market mismatch?. Journal of Marriage and Family.

[CR49] Nisén J, Martikainen P, Myrskylä M, Silventoinen K (2017). Education, other socioeconomic characteristics across the life course, and fertility among finnish men. European Journal of Population.

[CR50] Oláh LS, Bernhardt EM (2008). Sweden: Combining childbearing and gender equality. Demographic Research.

[CR51] Premfors R (1984). Analysis in politics: The regionalization of Swedish higher education. Comparative Education Review.

[CR52] Press JE (2004). Cute butts and housework: A gynocentric theory of assortative mating. Journal of Marriage and Family.

[CR53] Regnér, H., & Isacsson, G. (2008). Inkomstskillnader mellan par och singlar. *Är mönstren desamma för kvinnor och män*.

[CR54] Schytt E, Nilsen ABV, Bernhardt E (2014). Still childless at the age of 28 to 40 years: A cross-sectional study of Swedish women’s and men’s reproductive intentions. Sexual & Reproductive Healthcare.

[CR55] Silva EG (2016). The road to parenthood: Income and first births in Sweden. Finnish Yearbook of Population Research.

[CR56] Skirbekk V, Blekesaune M (2014). Personality traits increasingly important for male fertility: Evidence from Norway. European Journal of Personality.

[CR57] Skopek J, Schulz F, Blossfeld HP (2010). Who contacts whom? Educational homophily in online mate selection. European Sociological Review.

[CR58] Smits J (2003). Social closure among the higher educated: Trends in educational homogamy in 55 countries. Social Science Research.

[CR59] Tanturri ML, Mencarini L (2008). Childless or childfree? Paths to voluntary childlessness in Italy. Population and Development Review.

[CR60] Tanturri, M. L., Mills, M., Rotkirch, A., Sobotka, T., Takács, J., Miettinen, A., et al. (2015). *State*-*of*-*the*-*art report. Childlessness in Europe* (Vol. 32). Families and societies working paper series.

[CR61] Thomson E, Eriksson H (2013). Register-based estimates of parents’ coresidence in Sweden, 1969–2007. Demographic Research.

[CR70] Toulemon L, Pailhé A, Rossier C (2008). France: High and stable fertility. Demographic Research.

[CR62] Trimarchi A, Van Bavel J (2017). Education and the transition to fatherhood: The role of selection into union. Demography.

[CR63] Universitetskanslersämbetet [UKA]. (2016). *Kvinnor och män i högskolan. [Women and men in higher education].* Rapport 2016:16.

[CR64] Van de Kaa, D. J. (2001). Postmodern fertility preferences: from changing value orientation to new behavior. In R.A. Bulatao, Rodolfo & J.B Casterline (Eds.), *Global fertility transition. supplement to population and development review* (Vol. 27, pp. 290–331).

[CR65] Vincent-Lancrin Stéphan (2008). The Reversal of Gender Inequalities in Higher Education. Educational Research and Innovation.

[CR66] Wiik KA, Bernhardt E, Noack T (2010). Love or money? Marriage intentions among young cohabitors in Norway and Sweden. Acta Sociologica.

[CR67] Wiik KA, Dommermuth L (2014). Who remains unpartnered by mid-life in norway? Differentials by gender and education. Journal of Comparative Family Studies.

[CR68] Williams R (2012). Using the margins command to estimate and interpret adjusted predictions and marginal effects. Stata Journal.

